# Haziness Degree Evaluator: A Knowledge-Driven Approach for Haze Density Estimation

**DOI:** 10.3390/s21113896

**Published:** 2021-06-04

**Authors:** Dat Ngo, Gi-Dong Lee, Bongsoon Kang

**Affiliations:** Department of Electronics Engineering, Dong-A University, Busan 49315, Korea; datngo@donga.ac.kr (D.N.); gdlee@dau.ac.kr (G.-D.L.)

**Keywords:** haziness degree, haze density, haze-relevant feature, correlation analysis, analytical optimization

## Abstract

Haze is a term that is widely used in image processing to refer to natural and human-activity-emitted aerosols. It causes light scattering and absorption, which reduce the visibility of captured images. This reduction hinders the proper operation of many photographic and computer-vision applications, such as object recognition/localization. Accordingly, haze removal, which is also known as image dehazing or defogging, is an apposite solution. However, existing dehazing algorithms unconditionally remove haze, even when haze occurs occasionally. Therefore, an approach for haze density estimation is highly demanded. This paper then proposes a model that is known as the haziness degree evaluator to predict haze density from a single image without reference to a corresponding haze-free image, an existing georeferenced digital terrain model, or training on a significant amount of data. The proposed model quantifies haze density by optimizing an objective function comprising three haze-relevant features that result from correlation and computation analysis. This objective function is formulated to maximize the image’s saturation, brightness, and sharpness while minimizing the dark channel. Additionally, this study describes three applications of the proposed model in hazy/haze-free image classification, dehazing performance assessment, and single image dehazing. Extensive experiments on both real and synthetic datasets demonstrate its efficacy in these applications.

## 1. Introduction

Machine vision algorithms for high-level automatic visual recognition tasks in real-world systems appear to be unsuitable in adverse weather conditions owing to the absorption and scattering of incoming light waves. For example, a turbid atmosphere significantly reduces the visibility of captured scenes, posing severe problems for surveillance cameras or autonomous driving vehicles, and possibly causing undesired consequences. Haze removal algorithms have been used because revisiting deployed algorithms to consider the detrimental effects of the elements is seemingly impractical. In this context, image dehazing methods preprocess an input image/video stream to restore the scene radiance for subsequent algorithms. Nevertheless, because haze occurs occasionally, the unconditional removal of haze may become unfavorable when the input image is clear. Consequently, haze density estimation has attracted considerable interest from researchers over the past decade.

One of the first efforts to predict the hazy image’s visibility is to exploit multiple images that are captured under different weather conditions [1] or different polarization degrees [2]. However, these early models have been facing practical difficulties in obtaining sufficient images and configuring experimental equipment. Therefore, Hautiere et al. [3] proposed an automatic method for detecting the presence of haze and estimating the visibility distance using side geographical information that was obtained from an onboard camera. Although this method eliminates the requirement for multiple images, in practice it remains difficult to deploy. The main reason is the tradeoff between accuracy and algorithmic complexity. Creating an accurate three-dimensional model is a non-trivial task that is inappropriate for visibility estimation, which is supposed to be computationally efficient and compact. Conversely, using an approximated model similar to that proposed by Hautiere et al. [3] significantly affects the accuracy. Furthermore, this method is inapplicable to general hazy scenes because it is based on certain assumptions, for example, those regarding moving vehicles. Subsequently, Kopf et al. [4] presented a deep photography system to enhance the visibility of hazy images. Nevertheless, their method requires an existing georeferenced digital terrain and urban models to function correctly.

A more appealing approach is to exploit only a single hazy image; this method appears challenging, but it is highly promising for real-world applications. In this context, most dehazing algorithms utilize prior information regarding the scene radiance to compensate for the lack of external knowledge. Tan [5] assumed that the scene radiance had higher local contrast than the observed intensity. This assumption is suitable for estimating the scene albedo by maximizing the local contrast while assuming a smooth airlight; however, the recovered scene radiance tends to be overly saturated, which results in halo artifacts. He et al. [6] presented a pioneering study regarding the dark channel prior, which states that outdoor non-sky images possess extremely dark pixels in at least one color channel around local patches. Consequently, the dark channel prior can effectively estimate the raw transmission map, which inversely quantifies the haze density. He et al. [6] initially utilized soft matting to refine the raw transmission map and later sped up the refinement using a guided filter [7]. In contrast, Tarel and Hautiere [8] proposed a fast solution using an edge-preserving median of the median along a line filter. Although the algorithmic complexity is only a linear function of the pixel number, halo artifacts also affect the results. Kim et al. [9] developed a more sophisticated filtering technique, known as the modified hybrid median filter, to reduce the halo artifacts. Recently, Berman et al. [10] introduced the non-local haze-line prior, which postulates that a few tight clusters in the Red-Green-Blue (RGB) color space approximate to the haze-free image’s real color. However, a tradeoff between the restoration quality and run-time hinders the broad application of this prior.

Raikwar and Tapaswi [11] rearranged the atmospheric scattering model to estimate the transmission map based on the difference of minimum color channels in order to further improve visibility restoration. They adopted a bounding function to model this difference and exploited the regression technique to estimate the bounding function. Jiang et al. [12] proposed predicting the optical depth as a polynomial combination of haze-relevant features, in which sensitivity and error analyses were applied to reduce the model complexity. These two methods utilize synthetic datasets for estimation; hence, the domain shift problem may affect them when applied to real-world images. Wu et al. [13] formulated visibility restoration as a variational model for jointly achieving noise diminution and accuracy improvement. However, this method is computationally expensive and it may be affected by heterogeneous lighting conditions. Therefore, more efficient denoising methods [14,15] can be considered for reducing the computational complexity. Tang et al. [16] utilized another machine learning technique, which is known as random forest regression, to estimate the transmission map from a set of haze-relevant features. Similarly, Ngo et al. [17] optimized an objective function quantifying four haze-relevant features, including contrast energy, image entropy, local standard deviation, and normalized dispersion, to estimate the transmission map. Even though the restored visibility is impressive, the high computational cost precludes the broad application of these methods. Schechner and Averbuch [18] adopted adaptive regularization to develop a filtering approach for visibility restoration, but background noise affected the result in the distant region. Recently, Wu et al. [19] investigated the side effects of noise on visibility estimation. Subsequently, they proposed utilizing the interleaved cascade of shrinkage fields for noise diminution in the joint recovery of the scene radiance and transmission map. However, this method is also computationally expensive.

Furthermore, deep neural networks can be exploited to predict the haze density and scene radiance. Cai et al. [20] presented the first attempt to estimate the transmission map from a single image while using a three-layer convolutional neural network known as DehazeNet. The first layer extracts haze-relevant features, while the second layer processes these features at different scales to achieve spatial invariance. The last layer combines the results in a nonlinear manner to estimate the transmission map. However, DehazeNet does not demonstrate impressive performance because of its shallow architecture and simple learning strategy. Being inspired by DehazeNet, Li et al. [21] developed a lightweight all-in-one dehazing network (AOD-Net) for estimating the transmission map and atmospheric light in a unified manner. This type of estimation allows for the two latent variables to refine each other, consequently reducing the reconstruction error. Zhang and Tao [22] leveraged the compact architecture of the AOD-Net and the multiscale image fusion to design the FAMED-Net. This sophisticated network undoubtedly outperforms the AOD-Net in the visibility restoration task. It is also noteworthy that the AOD-Net and FAMED-Net can attain real-time processing when running on graphics processing unit platforms, which opens up a promising dimension toward deploying deep neural networks on edge devices. Huang et al. [23] devised a dual architecture comprising restoration and detection networks for the joint learning of three tasks: visibility enhancement, object classification, and object localization. However, this dual network is costly in terms of computational resources. Recent studies leveraged efficient encoder—decoder frameworks and more sophisticated loss functions to improve the estimation accuracy. Li et al. [24] exploited the encoder–decoder framework to develop a task-oriented network for haze removal, a refinement network for haze residual compensation, and a fusion network for fusing the previous two networks’ results. They also employed a loss function consisting of the mean absolute error, total variation, and dual composition losses.

The generative adversarial network (GAN), which is one of the most interesting technologies in computer science, can also be used to predict the scene radiance in hazy weather. Li et al. [25] presented a conditional GAN to mitigate unstable learning processes in GANs. Meanwhile, Pan et al. [26] developed a physics-based GAN to solve various ill-posed image restoration problems. Nevertheless, all of the deep-learning-based models share a common lack of complete and reliable training datasets for two main reasons: the sheer impracticality of capturing the same scene under different weather conditions and the unreliable performance of current depth cameras. Consequently, researchers have hitherto utilized synthetic datasets, in which hazy images or depth maps are synthesized from collected haze-free images or random distributions, respectively. This deficiency gives rise to the domain shift problem. Ignatov et al. [27] pioneered an effort to address this problem by loosening the strict requirement for paired datasets of supervised learning. In this context, they utilized two GANs that corresponded to forward and inverse mappings. The results generated by the forward GAN are converted back to the input domain by the inverse GAN, and the content consistency loss is exploited to ensure that the re-generated results exhibit similar characteristics as input images. Additionally, the forward GAN’s results are discriminated from the true data distribution on the basis of color and textual information. This innovative work enables network training using an unpaired dataset.

Previously, image fusion is a viable alternative for restoring the scene visibility in poor weather. This scheme yields a single image from several images, which can be generated from a sole input or captured from different cameras. Image dehazing in this manner offers considerable advantages, for example, few patch-based artifacts and a fast processing time. These benefits are attributable to the pixel-wise operation and the elimination of transmission map estimation. Ancuti et al. [28] exploited multiscale fusion for day and night-time single-image dehazing. The airlight is estimated in a patch-based manner using two different patch sizes because of the difference in the lighting conditions between the day and night-time scenes. Subsequently, two corresponding dehazed results, coupled with the discrete Laplacian of the original image, are fused to obtain the final result. The corresponding weight maps are derived from three essential features: image contrast, saturation, and saliency. Despite the satisfactory dehazing performance, up and down-sampling operations in the multiscale fusion hinder its broad application. Ngo et al. [29] recently demonstrated the insignificant performance gap between single and multiscale fusions, which favors the hardware implementation for real-time processing. It is also worth noting that Choi et al. [30] proposed an efficient method for haze density estimation, which is known as the fog aware density evaluator (FADE). The FADE predicts the haze density by exploiting the measurable deviations from the statistical regularities that were observed in real hazy and haze-free images. However, this metric is not in a normalized range, thereby resulting in difficulties in evaluating the haze density in general. Based on the comprehensive investigation when developing the FADE, Choi et al. [30] also devised a multiscale dehazing method, but it is computationally expensive.

Among all of the aforementioned methods, none of them are seemingly capable of removing haze judiciously. In this context, dehazing algorithms invariably attempt to remove haze from the input image, regardless of whether it is hazy or haze-free. Although researchers widely use the term "haze-free” to refer to clean images, it is noteworthy that these images are not completely free of haze. In practice, the atmosphere does contain microscopic aerosols, even in the clear weather, which gives rise to the inevitable existence of distant haze. However, this phenomenon is important for the human visual system to perceive depth information. Therefore, the absolute removal of haze may result in unnatural images, which may cause observers to lose the feeling of depth. This issue demands a visibility assessment tool quantifying the image’s haze density, which helps to classify hazy and haze-free images, and correspondingly perform image dehazing. In general, human subjective assessments are the most accurate method, despite being burdensome and non-repeatable. Accordingly, objective image quality assessment (IQA) algorithms are a possible alternative. Nevertheless, most of the existing IQA metrics require ground-truth references to assess visibility distortions; hence, they are inappropriate for the demanded task. In contrast, the FADE and optical depth prediction proposed by Jiang et al. [12] have been applied to visibility assessment from a single image; thus, they are used as benchmark methods in this study.

This study proposes a knowledge-driven approach for predicting haze density from a single image. It first explores several haze-relevant features and then selects three computationally efficient features based on a correlation and computation analysis. With these features, this study formulates an objective function for maximizing the scene radiance’s saturation, brightness, and sharpness while minimizing the dark channel. Afterwards, this study exploits analytical optimization to derive a closed-form expression of the proposed haziness degree evaluator (HDE). Additionally, it discusses three applications of HDE in hazy/haze-free image classification, dehazing performance assessment, and single image dehazing. Notably, the experimental results on hazy/haze-free image classification demonstrate that the proposed HDE is superior to the two aforementioned benchmark methods. The three main contributions of this study are as follows:

- This study presents a simple correlation and computation analysis to select image features that are haze-relevant and computationally efficient.
- With the selected features, this study formulates an analytically solvable objective function that simultaneously maximizes the scene radiance’s saturation, brightness, and sharpness, and minimizes the dark channel, which yields a closed-form formula for quantifying haze density from a single image.
- This study demonstrates that applying the proposed HDE to a particular task of hazy/haze-free image classification results in an accuracy of approximately 96%, which surpasses those of two benchmark metrics and human observers.

## 2. Preliminaries

### 2.1. Hazy Image Formation

The formation of hazy images in the atmosphere is a highly complex process involving several factors, such as diversity, orientation, and distribution of atmospheric turbidity [31]. Hence, the simplified Koschmieder model is widely used to describe the optical hazy image formation. When sunlight traverses the atmosphere to reach objects, atmospheric scattering and diffusion attenuate the constituent wavelengths, which results in an additive distortion that is known as airlight. Meanwhile, the light waves that are reflected from objects are affected by direct attenuation along the path to the camera’s aperture. Consequently, the Koschmieder model mathematically decomposes a hazy image into two components: direct attenuation and airlight, as shown in Equation (1).

(1) $$L\left(\lambda ,x\right)=L_{0}\left(\lambda ,x\right)exp\left[-\beta \left(\lambda \right)d\left(x\right)\right]+L_{\infty }\left(\lambda \right)\left\{1-exp\left[-\beta \left(\lambda \right)d\left(x\right)\right]\right\},$$

where *x* denotes the spatial coordinates of pixels in both the horizontal and vertical directions, λ represents the visible light wavelength, β denotes the atmospheric extinction coefficient, *d* the distance from the object to the observer, $L_{\infty }$ the mean sky irradiance, $L_{0}$ the scene irradiance, and *L* the image irradiance. The scene irradiance is a portion of the mean sky irradiance that is reflected from the object. In other words, $L_{0}$ can be expressed as $L_{\infty }$·*F*, where *F* is a dimensionless unit that denotes the reflectance factor. According to the International System of Units, λ and *d* are measured in meter (m), β reciprocal meter (m^−1^), and $L_{\infty }$, $L_{0}$, and *L* watts per square meter per meter (W·m^−3^).

The mapping from irradiance to image intensity, which is also known as the camera response function (CRF), depends on several factors, such as lens fall-off and the photosensor’s sensitivity. Despite such complexities, Grossberg and Nayar [32] discovered that the CRF is generally linear across the spatial dimensions of the image. Accordingly, it is convenient to set I(x)=L(λ,x), $J\left(x\right)=L_{0}\left(\lambda ,x\right)$, $A=L_{\infty }\left(\lambda \right)$, and t(x)=exp[−β(λ)d(x)] to simplify Equation (1).

**Figure 1:**
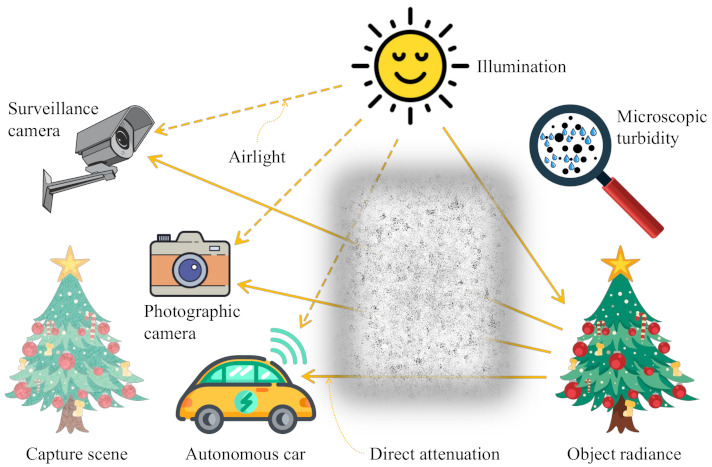
Optical hazy image formation.

(2) I(x)=J(x)t(x)+A[1−t(x)],

where *t* and A can henceforth be referred to as the transmission map and atmospheric light. The boldface representations of I, J, and A indicate their wavelength-dependent characteristics, whereas the dependency of *t* on wavelength is considerably weak, which results in the omission of wavelength in its expression. Accordingly, *t* is a single channel variable. In contrast, the boldfaced I, J, and A typically possess three channels that correspond to red, green, and blue wavelengths. Figure 1 illustrates the optical hazy image formation based on the simplified Koschmieder model. The turbid atmosphere comprising microscopic particles attenuates and scatters the incoming light waves, which causes direct attenuation and airlight represented by the corresponding terms Jt and A(1−t) that are shown in Equation (2), respectively. Hence, the captured scene exhibits some observable characteristics of hazy images, such as faint color, low contrast, and shifted luminance. This type of image degradation hinders the proper function of high-level automatic visual recognition algorithms being deployed in real-time systems. Therefore, a detailed investigation into haze-relevant features will provide useful insights for developing the HDE.

### 2.2. Haze-Relevant Features

Several haze-relevant features have been reported in the literature, such as those that were previously presented by Jiang et al. [12], Choi et al. [30], Min et al. [33], and Ancuti et al. [34]. This subsection then explores these studies and provides a brief description of features that are pertinent to predicting haze density from a single image. Readers that are interested in a comprehensive treatment are referred to the work conducted by Choi et al. [30]. First of all, the informative dark channel is considered. Based on extensive observations on outdoor non-sky haze-free images, He et al. [6] discovered that local image patches tend to possess dark pixels whose intensity is approximately zero because of objects’ diverse colors. In this context, at least one color channel must exhibit very low intensity, so that the object’s color can manifest itself. Conversely, hazy images exhibit a considerable increase in luminance as a result of the additive airlight, which results in the explicable absence of dark pixels. The same interpretation is applicable to the sky region that is characterized by bright colors. He et al. [6] define the dark channel $I^{dark}$ of an arbitrary image I, as follows:

(3) $$I^{dark}\left(x\right)=\min_{y\in \Omega (x)}\left[\min_{c\in \{R,G,B\}}I^{c}\left(y\right)\right],$$

where *c* denotes the color channel of I, Ω(x) represents the local patch centered at *x*, and *y* denotes the pixel coordinates within Ω(x). The channel-wise minimum operation $\min_{c\in \{R,G,B\}}\left(\cdot \right)$ yields a single-channel image; consequently, the spatial minimum filter $\min_{y\in \Omega (x)}\left(\cdot \right)$ yields a dark channel. It is noteworthy that, even though the minimum operators are commutative, the presented order is optimal. To simplify, the order reversal of the minimum operators presented in Equation (3) would approximately triple the computational load because filtering an RGB image requires three spatial filters.

One of the hazy image’s observable characteristics is low contrast due to the scattering and diffusion of reflected light in the atmosphere, as mentioned previously. Hence, contrast is an appropriate feature for haze detection and haze density estimation. Regarding the reliable measure of contrast, several indicators can be used, including the simple Michelson contrast or the complex contrast energy [30]. In this study, the contrast *C* is calculated in a patch-based manner as a variance of pixel intensities, as presented by Jiang et al. [12].

(4) $$C\left(x\right)=\sqrt{\frac{1}{3|\Omega (x)|}\sum_{y\in \Omega (x)}{∥I\left(y\right)-I\left(x\right)∥}^{2}},$$

where |Ω(x)| denotes the size of Ω(x) (for example, |5×5|=25), and ∥·∥ the L2 norm (or the Euclidean distance).

Two other observable characteristics of hazy images, pale color and shifted luminance, are pertinent to the saturation and brightness of the image. These two features are available in the Hue-Saturation-Value (HSV) color space, which is developed to resemble the way that humans perceive color-making attributes. Accordingly, they can be derived from the color space conversion, as shown in Equations (5) and (6) for normalized image data. An exciting observation emerging from the formulas of saturation *S* and value *V* is that their product SV becomes simpler, while a close correlation with haze density is retained.

(5) $$\begin{array}{ccc} S(x) & = & \frac{\max_{c\in \{R,G,B\}}I^{c}\left(x\right)-\min_{c\in \{R,G,B\}}I^{c}\left(x\right)}{\max_{c\in \{R,G,B\}}I^{c}\left(x\right)}, \end{array}$$

(6) $$\begin{array}{ccc} V(x) & = & \max_{c\in \{R,G,B\}}I^{c}\left(x\right), \end{array}$$

(7) $$\begin{array}{ccc} SV(x) & = & \max_{c\in \{R,G,B\}}I^{c}\left(x\right)-\min_{c\in \{R,G,B\}}I^{c}\left(x\right). \end{array}$$

In addition to the saturation in the HSV color space, the distribution of image pixels in the CIELab color space is exploited to measure the color attenuation of hazy images. The International Commission on Illumination defines this color space as a perceptually uniform space for detecting small color differences. Let ${\left[L\left(x\right),a\left(x\right),b\left(x\right)\right]}^{T}$ be the corresponding pixel values of ${\left[I^{R}\left(x\right),I^{G}\left(x\right),I^{B}\left(x\right)\right]}^{T}$ in the CIELab space. Hasler and Suesstrunk [35] measure the image chroma by converting Cartesian coordinates *a* and *b* to the cylindrical coordinate Ch. Similar to image saturation, chroma (also known as relative saturation) is significantly correlated with haze density, but it is unaffected by the image content. Jiang et al. [12] also observed a positive correlation between the variance of chroma and haze density. Hence, chroma Ch and its variance $\sigma_{Ch}^{2}$ are both informative and usable features for detecting the presence of haze and estimating haze density. Their corresponding formulas are as follows:

(8) $$\begin{array}{ccc} Ch(x) & = & \sqrt{a^{2}\left(x\right)+b^{2}\left(x\right)}, \end{array}$$

(9) $$\begin{array}{ccc} \sigma_{Ch}^{2}\left(x\right) & = & \sum_{y\in \Omega (x)}\omega \left(y\right){\left[Ch\left(y\right)-\mu_{Ch}\left(x\right)\right]}^{2}, \end{array}$$

(10) $$\begin{array}{ccc} \mu_{Ch}\left(x\right) & = & \sum_{y\in \Omega (x)}\omega \left(y\right)Ch\left(y\right), \end{array}$$

where ω(·) denotes the weighting function (for example, Gaussian or uniform) to calculate the mean $\mu_{Ch}$ and variance $\sigma_{Ch}^{2}$ values.

The aforementioned saturation and chroma denote the colorfulness of a color that is pertinent to its lightness. Another colorfulness measure, which quantifies the degree of difference between color and gray information, provides valuable insights into the haze density. Haseler and Suesstrunk [35] calculate this image colorfulness CF in the opponent color space using the following formula:

(11) $$CF\left(x\right)=\sqrt{\sigma_{rg}^{2}\left(x\right)+\sigma_{yb}^{2}\left(x\right)}+0.3\sqrt{\mu_{rg}^{2}\left(x\right)+\mu_{yb}^{2}\left(x\right)},$$

where rg and yb denote the red-green and yellow-blue channels, respectively. These two components are derived from an observed RGB image I, as follows:

(12) $$\begin{array}{ccc} rg(x) & = & I^{R}\left(x\right)-I^{G}\left(x\right), \end{array}$$

(13) $$\begin{array}{ccc} yb(x) & = & 0.5\left[I^{R}\left(x\right)+I^{G}\left(x\right)\right]-I^{B}\left(x\right). \end{array}$$

Additionally, an apparent loss of textual information due to atmospheric scattering affects the hazy image. Accordingly, its sharpness and details have diminished significantly. Based on this observation, the image entropy IE and image brightness variance $\sigma_{I}^{2}$ (also known as sharpness) are exploited to detect haze presence and estimate haze density. These statistical features are derived from a grayscale image $I^{gray}$, as shown in Equations (14) and (16), where $h_{y}$ denotes the grayscale intensity of the pixel and $p(h_{y})$ represents the corresponding probability of $h_{y}$ estimated from the normalized histogram.

(14) $$\begin{array}{ccc} \sigma_{I}^{2}\left(x\right) & = & \sum_{y\in \Omega (x)}\omega \left(y\right){\left[I^{gray}\left(y\right)-\mu_{I}\left(x\right)\right]}^{2}, \end{array}$$

(15) $$\begin{array}{ccc} \mu_{I}\left(x\right) & = & \sum_{y\in \Omega (x)}\omega \left(y\right)I^{gray}\left(y\right), \end{array}$$

(16) $$\begin{array}{ccc} IE(x) & = & -\sum_{y\in \Omega (x)}p\left(h_{y}\right)log_{2}\left[p\left(h_{y}\right)\right]. \end{array}$$

The last haze-relevant feature presented herein is the hue disparity that was proposed by Ancuti et al. [34] to generalize the dark channel approach of He et al. [6]. They define hue disparity HD as the absolute difference between hue values of the observed image I and its semi-inverted image $I_{si}$, as shown in Equation (17), where superscript *H* denotes the hue channel in the HSV color space and cmax(·) represents the channel-wise maximum operator. The dark channel approach can result in an inaccurate estimate of the transmission map in the sky region; therefore, the semi-inverted image is used in the hue disparity approach, as mentioned in Section 1.

(17) $$\begin{array}{ccc} HD(x) & = & |I^{H}\left(x\right)-I_{si}^{H}\left(x\right)|, \end{array}$$

(18) $$\begin{array}{ccc} I_{si}\left(x\right) & = & cmax[I(x),1-I(x)]. \end{array}$$

**Figure 2:**
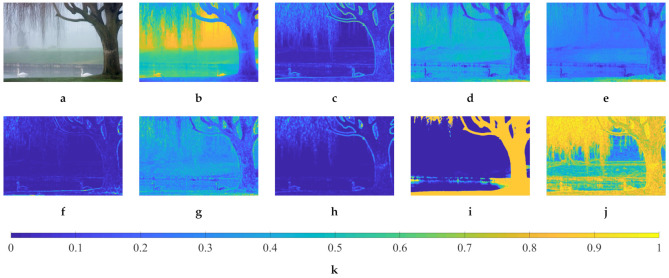
An illustration of a hazy image and its corresponding haze-relevant features. (**a**) Hazy image, and its (**b**) dark channel, (**c**) contrast, (**d**) saturation × value, (**e**) chroma, (**f**) variance of chroma, (**g**) colorfulness, (**h**) sharpness, (**i**) hue disparity, (**j**) image entropy, and (**k**) reference color bar.

Figure 2 demonstrates a real hazy image and its corresponding haze-relevant features. The feature values are min-max normalized, and Figure 2k depicts the reference color bar. It is observed that the hazy image shown in Figure 2a is comprised of three regions with mild, moderate, and dense haze. Among the nine features, the dark channel and image entropy presented in Figure 2b,j exhibit a close correlation to perceptual haze density, and they are on two opposite sides. The dark channel is directly proportioned to haze density, whereas image entropy manifests the inverse proportion. A correlation also exists between the remaining features and haze density, although it is not as evident as the previous two features. However, it is noteworthy that there currently does not exist an ideal haze-relevant feature that correlates perfectly with haze density. Accordingly, individual features may break down in certain circumstances, and therein lies the need for their mutual combinations. For example, the dark channel has incorrectly treated the white swans as densely hazy objects, owing to their bright appearance. Therefore, the image sharpness can be used to signify that they are informative objects.

Moreover, because nine features characterize the image’s three main aspects, including contrast, colorfulness, and sharpness, Figure 2 shows that some features are relatively similar to one another. For example, the dark channel is quite analogous with hue disparity and image entropy, because investigations on these three features result in a similar distinction between hazy regions. Similarly, there is a resemblance between the product of saturation and value, chroma, chroma’s variance, and colorfulness. Meanwhile, sharpness resembles local contrast to a moderately high degree. This observation, coupled with this study’s ultimate objective of deriving the proposed HDE’s closed-form expression, lies the motivation for the correlation and computation analysis shown in Section 3.3.

**Figure 3:**
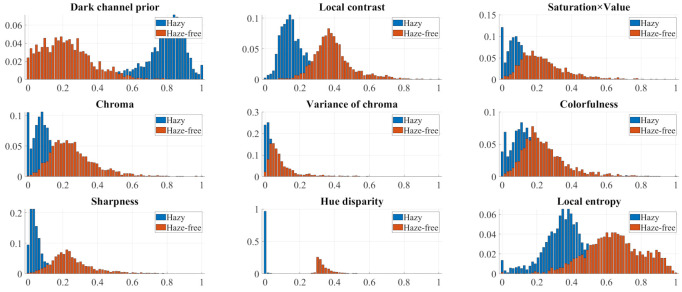
The normalized histograms of nine haze-relevant features. The horizontal axis is the normalized feature value, and the vertical axis is the pertinent frequency of occurrence.

Figure 3 illustrates the normalized histograms of all nine haze-relevant features that were calculated on both real and synthetic datasets, which are described in Section 3.2. Haze-free images contain a certain amount of haze at a distance for humans to perceive depth, as mentioned in Section 1. Accordingly, the features’ histograms exhibit various degrees of overlap depending on each feature’s sensitivity to haze. However, all of the features are, on the one hand, exploitable for detecting haze presence and estimating haze density. On the other hand, it is not recommended to utilize all of them because some features are not differentiable and they may result in a seemingly intractable objective function. Hence, in the upcoming section, a correlation and computation analysis for reducing the number of haze-relevant features will be presented. This simple analysis exploits the correlation between features and considers their computational complexity to yield easily differentiable features for formulating the objective function. Maximizing this function results in an optimal transmission map, which is inversely proportional to haze density and can be used to calculate the proposed HDE.

## 3. Haziness Degree Evaluator

The proposed HDE quantifies the haze density from a single image based on haze-relevant features, which characterize the image contrast, colorfulness, and sharpness, as described in Section 2.2. It is necessary to devise an analytically solvable objective function because this study aims to derive the HDE’s closed-form formula. Hence, this study analyzes the correlation between features and examines their calculation to draw three computationally efficient feature that will be used to formulate the objective function. This section begins by framing essential steps to derive the HDE. It then introduces the employed datasets, the feature selection scheme, the analytically solvable objective function, and the HDE’s closed-form formula. Finally, it concludes by discussing the necessity of using multiple haze-relevant features to derive the HDE.

### 3.1. Overview of HDE Derivation

**Figure 4:**
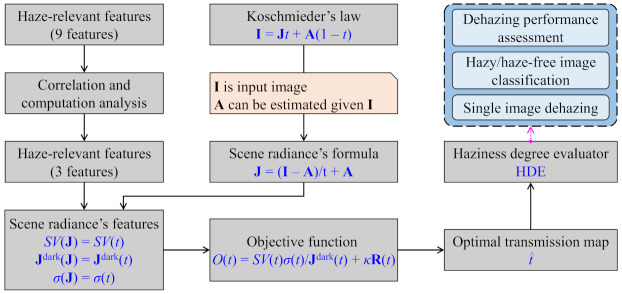
An overview of the proposed haziness degree evaluator’s derivation.

Figure 4 illustrates the HDE’s derivation from nine haze-relevant features, which are discussed in Section 2.2 based on the work of Jiang et al. [12], Choi et al. [30], Min et al. [33], and Ancuti et al. [34]. Because these features mutually characterize the image’s fundamental aspects, such as contrast, colorfulness, and sharpness, this study first analyzes their correlation and computation to reduce the number of employed features. This analysis step results in three features—dark channel, the product of saturation and value, and sharpness—that are haze-relevant and computationally inexpensive, as depicted in Figure 4. Meanwhile, the scene radiance’s formula can be obtained by rearranging Equation (2). Additionally, the fact that the sole input image suffices for estimating the atmospheric light lowers the number of unknowns to one, which lays the dependency of scene radiance on the transmission map. This dependency is exploited when calculating the aforementioned three features of scene radiance, which leads to their corresponding dependency on the transmission map. The objective function that is derived from those features is then not an exception. Optimizing this objective function results in the optimal transmission map, which is used to calculate the proposed HDE. In addition, Section 4 discusses three HDE-based applications, including hazy/haze-free image classification, dehazing performance assessment, and single image dehazing.

### 3.2. Employed Datasets

Datasets comprising hazy and haze-free images are necessary for calculating the normalized histograms shown in Figure 3, as discussed in Section 2.2, and calculating the correlation coefficients between haze-relevant features in the correlation and computation analysis. Real and synthetic datasets are both used in this study for a thorough evaluation of diverse images. IVC [36], O-HAZE [37], I-HAZE [38], FINEDUST [17], 500IMG [39], and Dense-Haze [40] are the real datasets considered. IVC consists of 25 hazy images depicting various scene types, including indoor/outdoor spots, daytime/night-time, landscapes, humans, and animals. O-HAZE comprises 45 pairs of hazy/haze-free outdoor images, whereas I-HAZE comprises 30 pairs of hazy/haze-free indoor images. Ancuti et al. [40] thereafter presented Dense-Haze, which is a larger dataset made up of 55 pairs of hazy/haze-free indoor and outdoor images. While the aforementioned datasets are widely publicized, FINEDUST and 500IMG are self-collected ones utilized in our previous studies. FINEDUST’s 30 constituent images are affected by notorious fine dust or yellow dust, and 500IMG’s 500 constituent images are haze-free images with a wide coverage of scene types.

This study also employs the FRIDA2 [41] and D-HAZY [42] datasets, which exemplify the main types of synthetic images. FRIDA2 comprises 66 ground-truth road-scene images that are generated by SiVIC™ software. From these images, the Koschmieder model and its variants are exploited to generate four sets of 66 hazy images (that is, 264 hazy images in total), including homogeneous, heterogeneous, cloudy homogeneous, and cloudy heterogeneous sets. Meanwhile, 1472 hazy indoor images in D-HAZY are derived from the corresponding real haze-free images. In this context, the requisite scene depths in the Koschmieder model are captured using the Kinect camera. Table 1 provides a summary of all eight datasets that were employed in this study, where NA stands for not available.

### 3.3. Correlation and Computation Analysis

In terms of statistical significance, correlation reflects the relationship between two variables, and several metrics are available for measuring the degree of correlation. Among them, the Pearson coefficient [43] appears to be the most prevalent. This coefficient is sensitive to a linear relationship, and its formula is as follows:

(19) $$\rho_{QZ4pt}=\frac{\sum_{i=1}^{n}\left(q_{i}-\bar{q}\right)\left(z_{i}-\bar{z}\right)}{\sqrt{\sum_{i=1}^{n}{\left(q_{i}-\bar{q}\right)}^{2}\sum_{i=1}^{n}{\left(z_{i}-\bar{z}\right)}^{2}}},$$

where (Q,Z) denotes the pair of variables considered, $\left(q_{i},z_{i}\right)$ represents one of *n* measurements, and $\left(\bar{q},\bar{z}\right)$ denotes the sample means of (Q,Z). The Pearson correlation coefficient $\rho_{QZ}$ ranges from −1 to 1, where 1 (or −1) signifies a perfect linear relationship (or an inverse one) and 0 signifies no association between (Q,Z). The correlations between the haze-relevant features are noticeable, as shown in Figure 3. Every feature, except the dark channel, exhibits a positive correlation with one another. Accordingly, this study utilizes the absolute value of the Pearson correlation coefficient for ease of expression.

**Figure 5:**
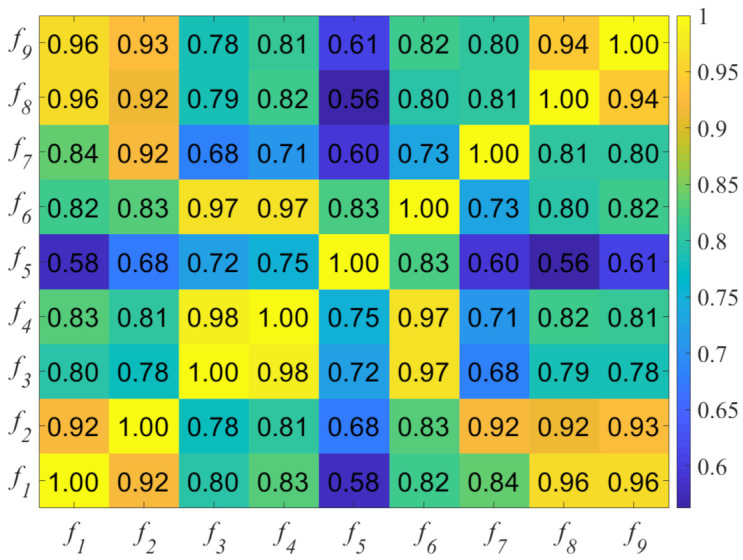
The absolute values of the Pearson correlation coefficients between haze-relevant features.

Figure 5 shows the correlation values that are calculated from the hazy images of employed datasets, where Table 2 shows each feature identification (ID) and its corresponding description. The computation follows the procedure that was presented by Choi et al. [30], according to which this study first divides hazy images into patches and then calculates the patch-based feature values. However, haze does not obscure all the patches within a hazy image; some of them may be haze-free, notably those that are close to the camera. Therefore, this study only selects representative patches based on the mean feature values for correlation computation. Regarding the dark channel ($f_{1}$), Figure 5 illustrates that it is highly correlated with the local contrast ($f_{2}$), hue disparity ($f_{8}$), and local entropy ($f_{9}$); hence, it is suggested that only one among the four features is adequate. Equations (3), (4), (16), and (17) demonstrate that the calculation of the dark channel ($f_{1}$) is the least involved. In particular, Equation (4) requires three squaring operations for individual pixels within the local patch and a square root of the accumulated square. Equation (16) requires the construction of a local normalized histogram, a logarithm operation, and an accumulation over the local patch. Equation (17) requires a channel-wise maximum operation, followed by a conversion to the HSV color space. By contrast, Equation (3) only requires two minimum operations. Therefore, this investigation supports selecting the dark channel ($f_{1}$) among the local contrast ($f_{2}$), hue disparity ($f_{8}$), and local entropy ($f_{9}$).

**Figure 6:**
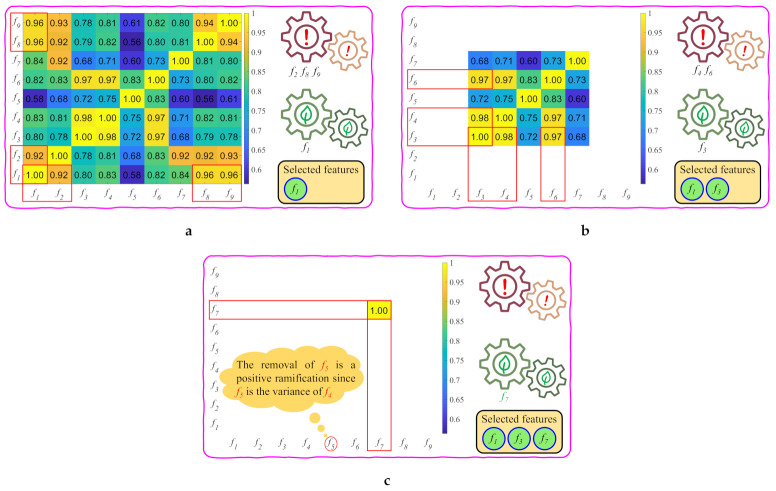
Illustration of the correlation and computation analysis for feature selection: (**a**) the first round, (**b**) the second round, and (**c**) the third round.

After performing the aforementioned investigation, five features remain, which is, $f_{i}$, where i∈Z∩[3,7]. This representation signifies that *i* is an element of the set intersection of the integers with the interval between three and seven. The investigation is now proceeding with the remaining features. Figure 5 demonstrates that the product of saturation and value ($f_{3}$) is closely correlated to chroma ($f_{4}$) and colorfulness ($f_{6}$). Equations (7), (8), and (11) then support selecting $f_{3}$ as the second usable feature. The calculation of chroma ($f_{4}$) and colorfulness ($f_{6}$) is considerably complicated, because it involves color space conversion, squares, square roots, means, and variances. By contrast, calculating the product of saturation and value ($f_{3}$) only involves channel-wise minimum and maximum operations. Furthermore, the variance of chroma ($f_{5}$) can also be excluded for the elimination of chroma ($f_{4}$). Consequently, one feature remains, which is, sharpness ($f_{7}$), which correlates with the computationally expensive local contrast ($f_{2}$). Hence, three features are selected after the correlation and computation analysis, including the dark channel ($f_{1}$), product of saturation and value ($f_{3}$), and sharpness ($f_{7}$). Because this study utilizes a considerably large number of images for investigating haze-relevant features, it can be postulated that the result of the correlation and computation analysis holds for all images in general. Figure 6 visually summarizes this subsection for ease of understanding. The red rectangles highlight the features that were investigated in each round, and the selected features are shown in the bottom-right corner. In the upcoming subsection, these three haze-relevant features are used to formulate an objective function to obtain the closed-form formula for the proposed HDE.

### 3.4. HDE Formula via Analytical Optimization of Objective Function

In the simplified Koschmieder model, the transmission map is inversely proportional to the haze density of the image. Hence, this study formulates an objective function that is based on the selected haze-relevant features, including the dark channel, product of saturation and value, and sharpness. Subsequently, this objective function is optimized to determine the optimal transmission map, which can be used to devise the HDE’s closed-form formula.

Firstly, rearranging Equation (2) results in the formula for the scene radiance as follows:

(20) $$J\left(x\right)=\frac{I(x)-A}{t(x)}+A,$$

where *t* is in the range (0,1] to prevent a division by zero. This value range is also directly derivable from the definition $t\left(x\right)=exp\left[-\beta (\lambda )d(x)\right]$ presented in Section 2.1 as the scene depth ranges from zero to infinity. Theoretically, the transmission map can become zero when the scene depth approaches infinity. However, because the current imaging technology is unable to capture image data at infinity, the transmission map only takes on values that are between (0,1].

Two postulates concerning the transmission map and atmospheric light are introduced before extracting haze-relevant features of the scene radiance. Because the transmission map is depth dependent, it is generally smooth, except for discontinuities, such as the objects’ contours. Hence, the first postulate is that the transmission in a local patch is constant, which is, $\min_{y\in \Omega (x)}t\left(y\right)=t\left(x\right)$. Additionally, atmospheric light is typically the brightest image pixel, which results in an insignificant difference between its constituent channels. Therefore, the second postulate is $A^{R}=A^{G}=A^{B}=A$. The plain symbol *A* is used herein to denote atmospheric light to conform with the second postulate, which simplifies a three-channel variable into a single channel variable. Using these two assumptions, coupled with the linearity of Equation (20), the dark channel, product of saturation and value, and sharpness of the scene radiance are as follows (it is noteworthy that the spatial coordinates are omitted for ease of expression):

(21) $$\begin{array}{ccc} J^{dark} & = & A-\frac{A-I_{m\Omega }}{t}, \end{array}$$

(22) $$\begin{array}{ccc} SV(J) & = & \frac{I_{mc}}{t}, \end{array}$$

(23) $$\begin{array}{ccc} \sigma_{J}^{2} & = & \frac{\sigma_{I}^{2}}{t^{2}}, \end{array}$$

where $I_{mc}=\max_{c\in \{R,G,B\}}I^{c}-\min_{c\in \{R,G,B\}}I^{c}$ and $I_{m\Omega }=\min_{y\in \Omega }\left(\min_{c\in \{R,G,B\}}I^{c}\right)$. Details on the derivation of the above equations can be found in Appendix A. Given the input image I, the atmospheric light *A* can be easily obtained using the quad-tree decomposition algorithm that was proposed by Park et al. [44]. The transmission map *t* is then an only unknown in Equations (21)–(23). Accordingly, it is convenient to regard the three features above as functions of *t*, which is, $J^{dark}\left(t\right)$, SV(t), and $\sigma_{J}^{2}\left(t\right)$. Moreover, utilizing $\sigma_{J}$ instead of $\sigma_{J}^{2}$ is highly beneficial in the subsequent optimization. Accordingly, the devised objective function, O(t), appears in Equation (24), where R(t) and κ are the regularization term and parameter, respectively. κR(t) is used to introduce adjustment ability to the transmission map that optimizes the objective function. SV(t) and $\sigma_{J}\left(t\right)$ are inversely proportional to the haze density, whereas $J^{dark}\left(t\right)$ exhibits the opposite relationship, as discussed in Section 2.2. Therefore, the objective function’s formula is explicable, because maximizing O(t) is similar to maximizing $SV\left(t\right)\sigma_{J}\left(t\right)$ while minimizing $J^{dark}\left(t\right)$.

(24) $$O\left(t\right)=\frac{SV\left(t\right)\sigma_{J}\left(t\right)}{J^{dark}\left(t\right)}+\kappa R\left(t\right).$$

Furthermore, the criterion for selecting the regularization term is that the optimization of O(t) is non-demanding and analytically solvable. Hence, this study selects R(t)=1/t. Subsequently, substituting Equations (21)–(23) into Equation (24) results in the following equation:

(25) $$O\left(t\right)=\frac{I_{mc}\sigma_{I}}{t(At-A+I_{m\Omega })}+\frac{\kappa }{t}.$$

Setting the first derivative of O(t) to zero while ensuring that the second derivative is negative yields the optimal transmission map, which is denoted as $\hat{t}$.

(26) $$\hat{t}=1-\frac{1}{A}\left[I_{m\Omega }+B-\sqrt{B(B-A+I_{m\Omega })}\right],$$

where

(27) $$\begin{array}{ccc} B & = & \frac{I_{mc}\sigma_{I}}{\kappa },\kappa \neq 0, \end{array}$$

(28) $$\begin{array}{ccc} \kappa & \leq & \frac{I_{mc}\sigma_{I}}{A-I_{m\Omega }}. \end{array}$$

Using the optimal transmission map $\hat{t}$, the proposed HDE can be calculated, as follows:

(29) $$\mathrm{HDE}=\frac{1}{\left|\Psi \right|}\sum_{\forall x\in \Psi }\left[1-\hat{t}\left(x\right)\right],$$

where Ψ denotes the entire image domain and |Ψ| is the total number of image pixels. The HDE exhibits the opposite trend because $\hat{t}$ is inversely proportional to the haze density. In addition, the HDE values are in the range [0,1), wherein the larger values indicate a higher degree of haziness (that is, thicker haze).

It is noteworthy that the HDE is derived from the optimal transmission map, which is, in turn, obtained through optimizing the scene radiance’s features. Thus, the proposed method bears some similarity to image dehazing algorithms that estimate the transmission map from a set of image features. The method that was developed by Tang et al. [16] is a prime example. It extracts haze-relevant features from a single hazy image and infers the corresponding transmission map utilizing the random forest regression technique. Although it is fundamentally similar to the proposed method, the key difference lies in the computational efficiency. Transmission map inference using random forest regression is extremely time-consuming, whereas the optimal transmission map presented in this study is conveniently calculated by a closed-form formula in Equation (26). Later on, Section 5 will demonstrate a run-time comparison between the proposed HDE and two benchmark methods for validating its computational efficiency.

Moreover, dehazing networks, such as DehazeNet [20], AOD-Net [21], and FAMED-Net [22], share a basic principle with the proposed HDE. Those models leverage the powerful representation capability of deep neural networks to estimate the transmission map or the *K* variable—a coalescence of the transmission map and atmospheric light. Accordingly, they can attain high estimation accuracy and spatial invariance, since the deep architecture allows for synthesizing robust high-level features from low-level features, which are generally extracted at the first hidden layer. The proposed HDE, in contrast, derives the optimal transmission map from three low-level features, which are already highly correlated to the haze density. Additionally, Equation (29) aggregates the optimal transmission map to obtain a single value for haze density estimation. Hence, high accuracy and spatial invariance are not the most prominent priorities. Consequently, the proposed HDE’s simplicity can facilitate its integration into other visibility restoration algorithms.

### 3.5. Necessity of Using Multiple Haze-Relevant Features to Derive the HDE

Visibility restoration from a single image in hazy weather is an ill-posed problem, since the unknown outnumbers the observation. Consequently, researchers have conducted extensive studies into the underlying relationship between hazy and haze-free images. Virtually all of these studies center around the following idea:

- Observing hazy and haze-free images, investigating statistical measures to discover regularities, and relating them to one or several image features.
- Utilizing the discovered features to infer the requisites for scene radiance recovery.

Subsequently, the number of image features and the degree to which they correlate with the haze distribution are decisive factors in the restoration quality of the scene radiance. Generally, the more image features that an algorithm leverages, the higher performance it can attain. This statement is supported by the recently reported state-of-the-art performance of deep-learning-based dehazing networks (for example, FAMED-Net [22]). These networks are equipped with many hidden layers, which allows them to learn both the low-level and high-level image features in a statistically robust manner. Nevertheless, this powerful representation capability comes at the cost of a heavy computational burden.

The proposed HDE utilizes low-level handcrafted image features whose correlation with haze density has been observed in the literature to bridge the gap between computational complexity and delivered performance. Additionally, a simple correlation and computation analysis has been conducted to select only three differentiable and computationally efficient features. This selection step is for the ultimate objective of deriving a closed-form formula to calculate the HDE. It is also necessary to leverage more than one feature, since the employed features can mutually compensate for each other’s failures. For example, suppose that this study only utilizes the dark channel to formulate the objective function. In that case, the optimal transmission map (${\hat{t}}_{DCP}$) becomes identical to that presented by He et al. [6], as shown in Equation (30). Accordingly, it also inherits all of the well-known shortcomings of the dark channel prior.

(30) $${\hat{t}}_{DCP}=1-\frac{I_{m\Omega }}{A}.$$

**Figure 7:**
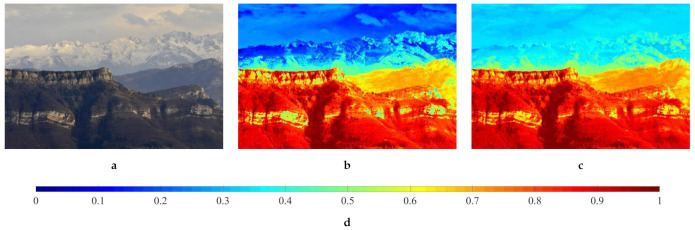
Illustration of a hazy image and its corresponding transmission map estimates. (**a**) Hazy image, and its (**b**) transmission map estimate based on the dark channel, (**c**) optimal transmission map derived in this study, and (**d**) reference color bar.

Figure 7 illustrates a real hazy image and its corresponding transmission map estimates using Equations (26) and (30) to validate the efficacy of utilizing multiple image features. In this figure, transmission map estimates are displayed with a different color map, as compared with Figure 2, depicting haze-relevant features, in order to avoid confusion. It is observed that Figure 7a comprises the distant snowy mountains and cloudy sky. Consequently, Equation (30) incorrectly estimates these regions as densely hazy, being represented by low values according to the reference color bar that is shown in Figure 7d. Restoring the scene radiance using this transmission map estimate causes color distortion in the sky region, as demonstrated later in Section 4.3. The proposed HDE, in contrast, correctly estimates those regions as moderately hazy, as depicted in Figure 7c. This improved accuracy is attributed to the other two features: the product of saturation and value and the sharpness, and therein lies an explicable reason for the HDE’s derivation.

## 4. HDE-Based Applications

In a study on the effects of image degradation on object recognition, Pei et al. [45] discovered that the reduction in accuracy is proportional to the haze density. Therefore, image dehazing algorithms are beneficial to high-level automatic visual recognition tasks in adverse weather conditions. However, they may become unfavorable in the clear weather because untoward image artifacts are observable in this case. Accordingly, the proposed HDE can bring a new dimension to the existing dehazing algorithms due to its ability to quantify the image’s haze density. This valuable piece of information can equip those algorithms with the "haze awareness” capability, which enables them to selectively dehaze input images. Hence, this section discusses the hazy/haze-free image classification task using the proposed HDE and provides the experimental results to demonstrate its superiority in this application. Additionally, this section provides a discussion on using HDE as a quantitative assessment tool as well as demonstrating its dehazing capability.

### 4.1. Hazy/Haze-Free Image Classification

It is clear that haze exists in the image because the atmosphere is not entirely free of turbidity, as mentioned earlier in Section 1. In other words, the quantification of the image’s haze density using the proposed HDE results in a non-zero value. Therefore, it is essential to determine the decision value for the hazy/haze-free image classification task. In this context, a particular image is regarded as a hazy image if its HDE value is larger than the decision value, and vice versa. This study determines the decision value based on a trivial looping method. First, given the data Φ comprising hazy and haze-free images, this method calculates the corresponding HDE values and then organizes them into hazy and haze-free sets. After that, it initiates the decision value DV using the average of the mean HDE values of these two sets. Subsequently, it iteratively changes DV around its initial value and calculates the corresponding accuracy $ACC_{\Phi }$. Finally, it selects the DV value that results in the maximum $ACC_{\Phi }$, as described by the following expression.

(31) $$\underset{DV}{\mathrm{argmax}}ACC_{\Phi }.$$

In this study, the employed data Φ consists of eight datasets that were previously summarized in Table 1. The total numbers of hazy and haze-free images are then 1921 and 2168, respectively. These images include both real and synthetic scenes that cover a wide range of scenarios. Therefore, the diversity in image content, coupled with copious amounts of image data, supports using the decision value determined herein to classify hazy/haze-free images in general. Appendix B presents the corresponding experimental results and source code for reproducibility to avoid digression.

A brief review of terminologies and derivations, as presented by Chicco and Jurman [46], is discussed next as the preliminary stage of accuracy computation. In the hazy/haze-free image classification task, “hazy” is a positive class, and “haze-free” is a negative class. Accordingly, the condition positive *P* is the number of real positive cases in the data Φ. The true positive TP is the number of cases in which the proposed evaluator correctly predicts as positive (that is, hazy). Conversely, for positive cases, the false negative FN is the number of cases that the proposed evaluator incorrectly predicts to be negative. Similarly, the condition negative *N* is the number of real negative cases in the data Φ. The true negative TN is the number of cases in which the proposed evaluator correctly predicts as negative (that is, haze-free). The false positive FP is the number of cases that the proposed evaluator incorrectly predicts to be positive. Table 3 provides a summary of these terminologies and derivations.

The four outcomes that are listed in Table 3 correspond to the four probabilities, namely the true positive rate TPR, false negative rate FNR, true negative rate TNR, and false positive rate FPR. The following formulas are used to calculate the TPR, FNR, TNR, FPR, and accuracy $ACC_{\Phi }$ for data Φ. It is noteworthy that the data Φ comprise positive *P* and negative *N* conditions.

(32) $$\begin{array}{ccc} TPR & = & \frac{TP}{P}=\frac{\sum_{i\in P}\left[{\mathrm{HDE}}_{i}>DV\right]}{P}, \end{array}$$

(33) $$\begin{array}{ccc} FNR & = & \frac{FN}{P}=\frac{P-TP}{P}=1-TPR, \end{array}$$

(34) $$\begin{array}{ccc} TNR & = & \frac{TN}{N}=\frac{\sum_{i\in N}\left[{\mathrm{HDE}}_{i}\leq DV\right]}{N}, \end{array}$$

(35) $$\begin{array}{ccc} FPR & = & \frac{FP}{N}=\frac{N-TN}{N}=1-TNR, \end{array}$$

(36) $$\begin{array}{ccc} ACC_{\Phi } & = & \frac{TP+TN}{P+N}. \end{array}$$

In Equations (32) and (34), this study adopts Iverson’s convention [47], which encloses a true-or-false statement in brackets. The term [·] is one when the enclosed statement is true, and it is zero otherwise. In addition to the proposed HDE, the optical depth that was proposed by Jiang et al. [12] (henceforth referred to as DF for short) and the FADE [30] are also haze density evaluators. Accordingly, the aforementioned binary classification task is attainable using the DF or FADE. Specifically, the procedure for determining the decision value that results in the maximum classification accuracy is repeated, except that FADE and DF are used instead of HDE. Table 4 summarizes the classification results using three haze density evaluators, including the FADE, DF, and the proposed HDE. In Table 4, HDE${}_{\beta }$ denotes the proposed HDE at the current stage, because further effort will be expended to improve the classification accuracy. Although the HDE achieves the highest accuracy of 93.8%, it appears to be unimpressive when compared with the human accuracy of approximately 95%, which is obtained from the results of an ImageNet large-scale visual recognition challenge [48]. Hence, this study will present the image intensity emphasis for increasing the classification accuracy.

**Figure 8:**
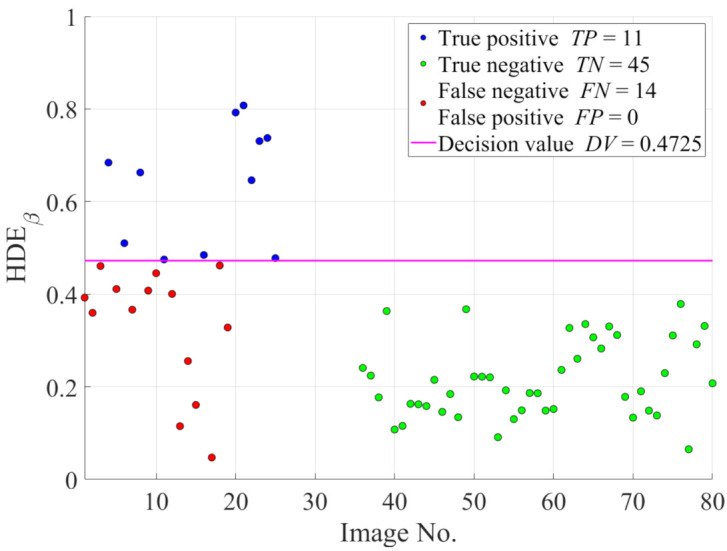
The scatter plot of the HDE${}_{\beta }$ values of images in the IVC and O-HAZE datasets.

In the follow-up investigation, only 25 hazy images from the IVC dataset and 45 haze-free images from the O-HAZE dataset are utilized to demonstrate the efficacy of image intensity emphasis in improving the classification accuracy for ease of presentation. Figure 8 illustrates the HDE values of these images as well as the decision value. For the two datasets, the total number of incorrectly predicted cases is 14. However, from Figure 8, it is noteworthy that the FN can decline if the HDE values increase and exceed the decision value. Therefore, revisiting Equation (26) provides insights into the method by which the HDE values can be increased, which is, modifying either the input image I or regularization parameter κ. Among these two options, because some restrictions are imposed on κ, the former appears to be more feasible and it results in a further investigation with regard to modifying *A*, $I_{m\Omega }$, $I_{mc}$, and $\sigma_{I}$.

Of all four possibilities, modifying *A* and $I_{mc}$ is ineffective, because *A* is typically the brightest pixel in the image, and $I_{mc}$ is the difference between two extreme channels. Hence, there remain two ways to increase the HDE values through increasing $I_{m\Omega }$ and decreasing $\sigma_{I}$. These two operations are then attainable while using channel-wise intensity emphasis, as shown below.

(37) $$I_{e}=I^{\gamma },$$

where 0<γ<1 denotes the emphasis strength and the subscript *e* stands for emphasis. Through this simple operation, the image intensity increases globally and, hence, the increase in $I_{m\Omega }$. Additionally, an increase in the image intensity causes the data to be closer to the mean, which results in a reduced $\sigma_{I}$.

**Figure 9:**
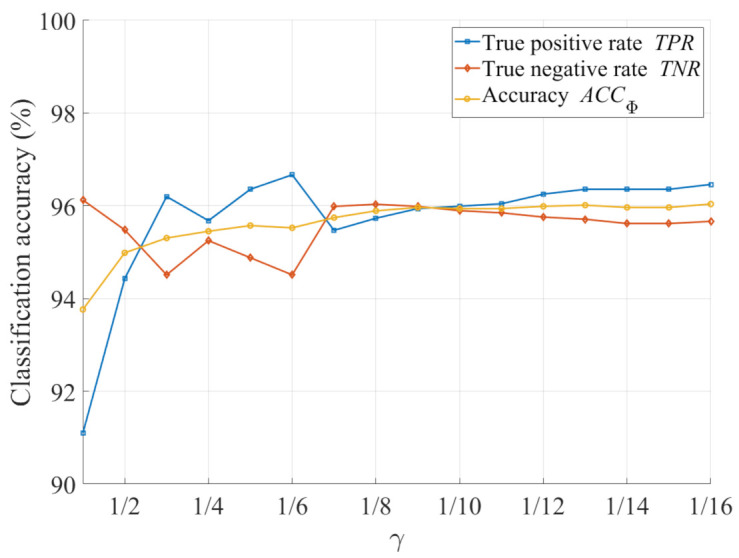
Classification accuracy as a function of γ.

**Figure 10:**
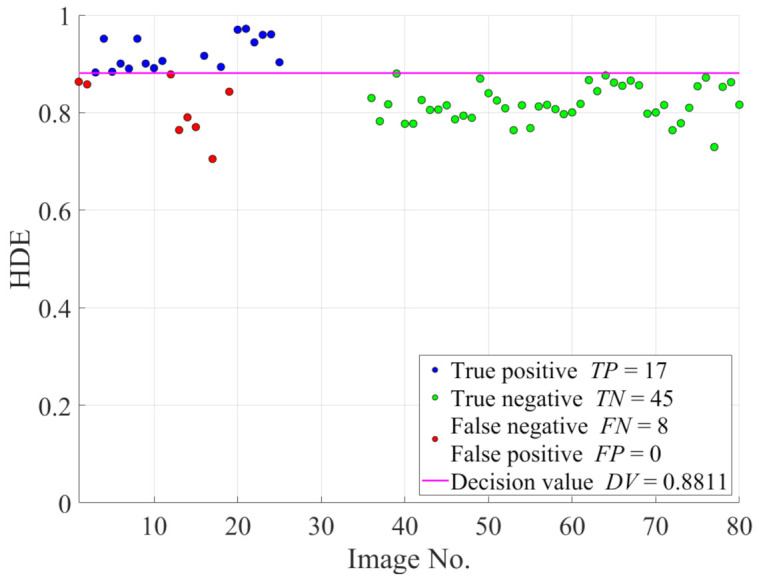
Scatter plot of the HDE values of images in the IVC and O-HAZE datasets after image intensity emphasis.

Figure 9 demonstrates the classification accuracy as a function of γ to provide insights into the empirical determination of the γ value. It is observed that the proposed HDE more accurately classifies hazy and haze-free images as γ decreases. In addition, there is no significant change in the classification accuracy after it reaches 96%. Therefore, although γ values from 1/8 to 1/11 appear to be acceptable, the value 1/9 is selected because it is virtually the point of intersection among the TPR, TNR, and $ACC_{\Phi }$. Figure 10 illustrates the new HDE values of the images in the IVC and O-HAZE datasets after performing image intensity emphasis. It is noteworthy that the decision value has been redetermined using Equation (31) to ensure the maximum accuracy. Hence, the number of incorrectly predicted cases has decreased significantly, which is, from 14 to 8, as depicted in Figure 10.

Table 5 shows the updated accuracy report for the hazy/haze-free image classification using the FADE, DF, HDE${}_{\beta }$, and the proposed HDE with image intensity emphasis. By virtue of this simple image processing technique, the proposed HDE has more accurately classified the hazy images while retaining an impressive TNR for haze-free images. Therefore, a considerable increase in the TPR (4.8%) has boosted the accuracy to 96%, which is superior to those of the FADE, DF, as well as human observers.

### 4.2. Dehazing Performance Assessment

An image’s HDE value lies in the range [0,1), and it is proportional to the haze density, as discussed in Section 3.4. Therefore, similar to other IQA metrics, the proposed HDE is exploitable in dehazing performance assessment, wherein lower HDE values signify a stronger dehazing power. Additionally, the proposed HDE offers a definite advantage over full-reference IQA metrics, in that it does not require ground-truth references for quantitative assessment. Hence, it is apposite in evaluating dehazing algorithms on general hazy scenes whose haze-free references are usually unavailable.

This section evaluates three typical dehazing algorithms that were proposed by He et al. [6], Tarel and Hautiere [8], and Zhu et al. [49] on the LIVE [30] and D-HAZY [42] datasets. The LIVE dataset consists of 500 hazy images and 500 unpaired haze-free images, while the D-HAZY dataset has been introduced earlier in Section 3.2. Although two datasets comprise hazy and haze-free images, this study only utilizes hazy images to assess the dehazing performance. On the LIVE dataset, the average HDE values that are summarized in Table 6 demonstrate that the algorithm proposed by He et al. [6] is the best performing method. The second best and third best are those proposed by Tarel and Hautiere [8] and Zhu et al. [49], respectively. When compared with the assessment using the FADE metric conducted by Galdran [50], our experimental results correctly reflect the actual dehazing performance. Specifically, two experiments are conducted on the same dataset, but with different assessment metrics. The results reported by Galdran [50] demonstrate that the method that was proposed by Tarel and Hautiere [8] is not usually the best method, despite the fact that the opposite is widely validated in the literature. The evaluation results that were provided by Ancuti et al. [42] are a prime example. They exploited a full-reference IQA metric, known as the structural similarity (SSIM) [51], to quantitatively assess dehazing algorithms on the D-HAZY dataset. The method that was proposed by He et al. [6] is superior to that proposed by Tarel and Hautiere [8] in terms of SSIM, as shown in the last row of Table 6. This result is consistent with our result on the D-HAZY dataset using the proposed HDE.

### 4.3. Single Image Dehazing

**Figure 11:**
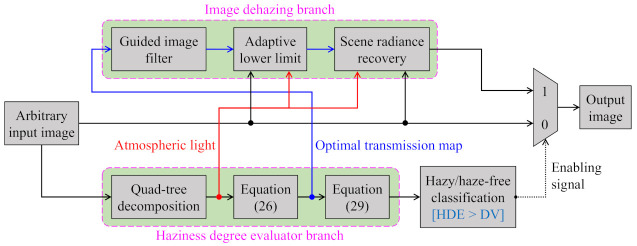
Block diagram of the HDE-based dehazing algorithm.

A byproduct of the proposed HDE, the optimal transmission map in Equation (26), is exploitable in single image dehazing. Figure 11 depicts the block diagram of the HDE-based dehazing algorithm for handling an arbitrary input image, regardless of whether it is hazy or haze-free. In this context, the aforementioned hazy/haze-free image classification task is invoked on the input image to obtain an enabling signal. This signal is zero when the input image is haze-free, and vice versa. It is then utilized to route the desired result to the output, which conforms with the following straightforward principle:

- The HDE-based algorithm by-passes the input image if it is haze-free.
- Otherwise, it outputs the result of the image dehazing branch.

Regarding the image dehazing branch, the transmission map and atmospheric light are two requisites for recovering the scene radiance. Fortunately, they can be obtained during the HDE’s calculation. This study utilizes the quad-tree decomposition algorithm [44] to estimate the atmospheric light from the single input image, as mentioned in Section 3.4. Equation (26), in turn, yields the transmission map, which further undergoes the guided-filtering-based refinement step [7]. Before recovering the scene radiance according to Equation (20), this study explicitly imposes an adaptive lower limit on the refined transmission map to ensure that the dehazed result is not black-limited. This problem occurs when the transmission map value is too small and, thus, Equation (20) can yield negative values, notably for the dark image regions. Those negative values are referred to as arithmetic underflow, and they are often limited to zero, and therein lies the occurrence of black pixels. Color distortion may become observable when the corresponding transmission map values are too small, even for other image regions. A majority of the existing algorithms in the literature have adopted a fixed lower limit to constrain the transmission map. For example, He et al. [6] and Zhu et al. [49], respectively, leveraged the lower limits of 0.1 and 0.05. However, the fixed lower limit is not a viable solution in a general case. Therefore, our previous work [52] proposed an adaptive lower limit to remedy this problem. The final transmission map (${\hat{t}}_{f}$) for the scene radiance recovery step is then expressed, as follows:

(38) $${\hat{t}}_{f}=min\left[GF\left(\hat{t}\right),t_{l}\right],$$

where min(·) denotes the element-wise minimum operation, GF(·) represents the guided image filtering operation, and $t_{l}$ denotes the adaptive lower limit. It is noteworthy that the input image is exploited as guidance in GF(·), and $t_{l}$ can be easily calculated given the input image and the atmospheric light. However, the complete formula of $t_{l}$ is relatively lengthy, and interested readers are referred to our previous work [52] for a full description.

Equation (20) can be modified to reflect the scene radiance recovery of the HDE-based dehazing algorithm, as presented below:

(39) $$J\left(x\right)=\{\begin{array}{cc} \frac{I(x)-A}{{\hat{t}}_{f}}+A & \mathrm{HDE}>DV\\ I(x) & \mathrm{HDE}\leq DV. \end{array}$$

It is worth recalling that this study has postulated that the difference between three constituent channels of the atmospheric light is negligible in Section 3.4. Thus, Equation (39) has utilized the plain symbol *A*.

**Figure 12:**
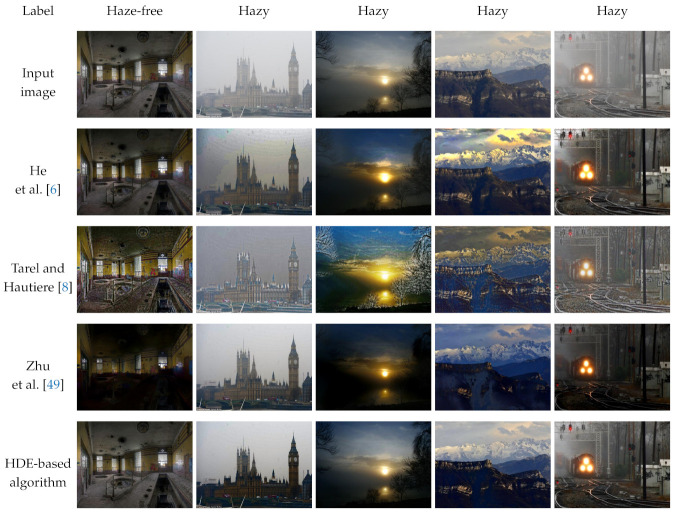
A qualitative comparison of the HDE-based dehazing algorithm with state-of-the-art methods on real images.

This study now presents the qualitative and quantitative assessments for evaluating the HDE-based dehazing algorithm in comparison with the aforementioned typical methods that are shown in Section 4.2. Because these algorithms generally produce satisfactory results, this study first qualitatively assesses their performance on images that may cause post-dehazing artifacts, as demonstrated in Figure 12. Regarding the haze-free image in the first column of Figure 12, benchmark methods are unaware of whether it is hazy or haze-free. Accordingly, they dehaze the haze-free image, which gives rise to untoward distortion, as observed in the results reported by Tarel and Hautiere [8] and Zhu et al. [49]. Meanwhile, the result presented by He et al. [6] is slightly darker than the haze-free input, which is attributed to its well-recognized performance for indoor images. However, on hazy images with a broad sky, the results reported by He et al. [6] suffer from color distortion because the dark channel prior does not hold for sky regions. The results that were published by Tarel and Hautiere [8] and Zhu et al. [49] also exhibit color distortion for the mountain image in the fourth column. Additionally, halo artifacts are observable in the results presented by Tarel and Hautiere [8], notably in the building image in the second column. In contrast, the HDE-based dehazing algorithm is equipped with hazy/haze-free discrimination ability. Therefore, it recognizes the haze-free image and skips the dehazing process. Regarding hazy images, it produces satisfactory results without any noticeable artifacts.

Additionally, Table 7 provides the results of a comparative evaluation with those three benchmark methods using full-reference and blind IQA metrics. For datasets with ground-truth references, such as D-HAZY, O-HAZE, and I-HAZE, the feature similarity index extended to color images (FSIMc) [53] and the tone-mapped image quality index (TMQI) [54] are leveraged to quantitatively assess the dehazed images. The FSIMc scores the image quality locally using two low-level features: phase congruency and image gradient magnitude. It then weights these scores using the phase congruency and averages the weighted scores to obtain a single score ranging between zero and unity. The higher this score, the greater degree to which the dehazed image resembles the ground-truth reference. Similarly, the TMQI also varies between zero and unity, in which the higher score is favorable in image processing tasks. However, the TMQI assesses the image quality based on the multiscale similarity index and the measure of naturalness.

The rate of new visible edges (*e*) and the quality of contrast restoration (*r*) are employed as blind IQA metrics for the dataset without ground-truth references, such as IVC [55]. They are calculated using the visible edges, which were originally invisible in the input image. These edges, in turn, are determined based on a pre-defined local contrast threshold. Consequently, the *e* and *r* metrics are prone to spike-like noises, such as halo artifacts. Hence, although higher *e* and *r* values are theoretically favorable, they should be considered in conjunction with a qualitative evaluation for a reliable assessment.

The HDE-based dehazing algorithm is the second-best in terms of *e* and *r*, whereas the best is the method that was proposed by Tarel and Hautiere [8], as demonstrated in Table 7. Nevertheless, the results that are presented in Figure 12 have shown that this method severely suffers from halo artifacts in fine detail, which contribute to its high *e* and *r* values. Therefore, the HDE-based dehazing method is considered to be the best on the IVC dataset. On other datasets, it can be observed that four methods exhibit comparative performance. Specifically, on the D-HAZY dataset, the method that was proposed by He et al. [6] demonstrates the best performance, agreeing with the evaluation results that were reported by Ancuti et al. [42]. Under the TMQI metric, the HDE-based dehazing method is ranked second, third, and second on the D-HAZY, O-HAZE, and I-HAZE datasets. Meanwhile, it is also ranked fourth, second, and first under the FSIMc metric. This quantitative assessment, coupled with the qualitative evaluation mentioned above, verifies the performance of the HDE-based dehazing method.

## 5. Discussion

**Figure 13:**
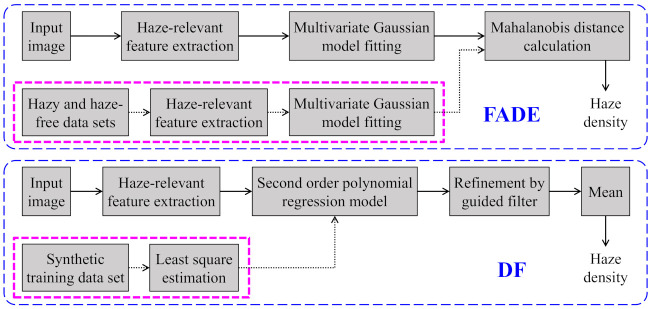
The block diagrams of two benchmark evaluators.

The proposed HDE is a knowledge-driven approach, which is, it does not require any training on collected data prior to its deployment. By contrast, the FADE and DF are data-driven approaches, wherein data collection for the pre-calculation of their local parameters is essential. Specifically, an offline calculation for obtaining the mean vectors and covariance matrices of the corresponding hazy and haze-free image corpora is indispensable because the FADE estimates the haze density based on the Mahalanobis distance in a haze-relevant feature space. Meanwhile, the DF estimates the haze density based on the optical depth, which is the output of a regression model whose parameters are derived from least-squares estimation on a synthetic training dataset. Figure 13 depicts the block diagrams of these two benchmark evaluators and highlights the offline calculation in pink. Conversely, the proposed HDE does not require any offline calculation. Instead, it estimates the haze density directly from a single input image and it is more computationally efficient and convenient.

Table 8 demonstrates the run-time comparison between three haze density evaluators. The experimental results tabulated therein are measured in the MATLAB R2019a environment, running on a computer with an Intel Core i7-9700 (3.0 GHz) CPU and 32 GB RAM. In relation to the FADE and DF, the aforementioned offline calculation does not affect the run-time, because it is performed in advance. Accordingly, the FADE and DF exhibit relatively fast processing speeds. However, they are still slower than the proposed HDE. On the one hand, the FADE’s time-consuming parts are haze-relevant feature extraction and Mahalanobis distance calculation. The former extracts as many as twelve features, despite the fact that some of them correlate with each other. Meanwhile, the latter is slow, owing to matrix manipulation. On the other hand, although the DF has reduced the number of features through sensitivity and error analyses, it is still not as fast as the HDE due to the use of mutual combinations between features in the regression model. In contrast, the HDE is the fastest method among the three evaluators. This high speed is attributed to the closed-form formula supporting haze density prediction from a single image.

**Figure 14:**
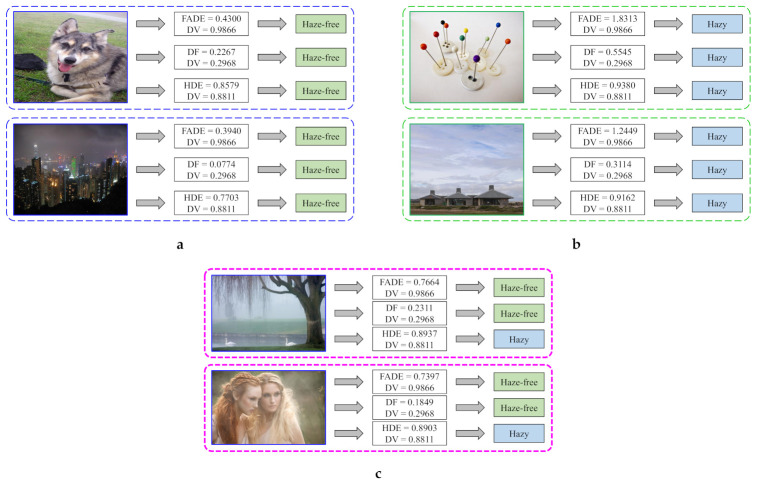
A comparison of the proposed evaluator with state-of-the-art evaluators: (**a**) false-negative cases, (**b**) false-positive cases, and (**c**) superior cases.

Nevertheless, the three evaluators share some common drawbacks, such as *FN*s and *FP*s, as illustrated in Figure 14a and Figure 14b, respectively. In Figure 14a, the FADE, DF, and HDE have incorrectly classified thin-haze and night-time images as haze-free images. In relation to the thin-haze image, it can be observed that the HDE value is close to the decision value. Because the classification of images whose HDE value is close to the decision value is ambiguous, the failure of the HDE is explicable. However, the same interpretation does not hold for the FADE and DF. Regarding the night-time image, incorrect classification is a typical shortcoming among three evaluators. One possible reason is that the atmospheric light estimate utilized in the HDE’s calculation does not reflect the heterogeneous illumination of night-time scenes. Therefore, it is determined that utilizing the local estimate of atmospheric light may be a viable solution. In this context, the local estimate can be obtained using the novel maximum reflectance prior, as proposed by Zhang et al. [56,57] for night-time image dehazing. However, because a more comprehensive investigation has to be done before discovering the exact reason, this failure in night-time scenes is left for future studies.

Similarly, the *FP* cases presented in Figure 14b demonstrate that all three evaluators have incorrectly classified haze-free images as hazy images. This failure occurs owing to the large sky region and smooth background. These haze-like regions pose a challenging problem for discriminating them from the actual hazy region. In that case, a thorough investigation into the image’s cumulative distribution function may provide useful insights. Moreover, leveraging semantic information may also be a viable alternative that is worthy of further investigation. These valuable pieces of information can be used to guide the final average pooling to produce a robust estimate. However, this issue also requires a more detailed investigation in future studies, similar to the *FN* case on the night-time image.

Finally, Figure 14c illustrates some cases where the proposed HDE is superior to the FADE and DF. It is clear that the two images that are depicted in Figure 14c are obscured a considerable amount of haze. However, the FADE and DF have incorrectly classified these two as haze-free images with a substantial degree of confidence, as represented by relatively large distances to the decision values. Conversely, the proposed HDE has yielded *TP*s and, hence, is superior to the FADE and DF.

## 6. Conclusions

This paper presented an HDE for haze density estimation from a single image. The proposed approach is knowledge-driven, as opposed to data-driven evaluators, such as the FADE and DF. Firstly, a simple correlation and computation analysis was presented to select image features that are highly pertinent to haze and are computationally efficient. An analytically solvable objective function, whose optimization is analogous to maximizing the image’s saturation, brightness, and sharpness, while minimizing the dark channel, was then formulated from these features. Optimizing this objective function resulted in an HDE’s closed-form formula. This paper also demonstrated three HDE-based applications, including hazy/haze-free image classification, dehazing performance assessment, and single image dehazing. In relation to the classification application, the experimental results showed that the proposed HDE achieved an impressive accuracy of 96%, outperforming the benchmark evaluators as well as human observers. Equipped with this superiority, the proposed evaluator can accurately quantify the image’s haze density; consequently, it can benefit the quantitative assessment of dehazing algorithms. Additionally, the proposed evaluator and its byproduct (that is, the optimal transmission map) can be exploited to improve dehazing algorithms’ performance in both hazy and clear weather conditions.

Nevertheless, a challenging problem arises when predicting the haze density of images under specific circumstances, for example, hazy night-time images or haze-free images containing a smooth background or a broad sky. This is attributable to the heterogeneous illumination of night-time scenes or the low-frequency constituent components of a smooth background or a broad sky. In addressing the former problem, leveraging the novel maximum reflectance prior information to obtain a spatially adaptive estimate of the atmospheric light might be a feasible solution. Meanwhile, a comprehensive investigation into the image’s cumulative distribution function and semantic information may provide helpful insights into addressing the latter problem. However, there are strict requirements for algorithmic complexity since haze density prediction and visibility restoration are widely considered preprocessing steps in high-level applications. Therefore, we will seek efficient and straightforward techniques to surmount those challenging problems in future studies.

## Figures and Tables

**Table 1:** Summary of the real and synthetic datasets employed in this study. NA stands for not available.

Dataset	Type	Hazy Images (#)	Haze-Free Images (#)	Ground Truth
IVC	Real	25	NA	No
FRIDA2	Synthetic	264	66	Yes
D-HAZY	Synthetic	1472	1472	Yes
O-HAZE	Real	45	45	Yes
I-HAZE	Real	30	30	Yes
FINEDUST	Real	30	NA	No
500IMG	Real	NA	500	No
Dense-Haze	Real	55	55	Yes

The “#” symbol denotes the number of images.

**Table 2:** A summary of haze-relevant features. ID stands for feature identification.

ID	Symbol	Description	Computation
$f_{1}$	$I^{dark}$	Dark channel	Equation (3)
$f_{2}$	*C*	Contrast	Equation (4)
$f_{3}$	SV	Saturation × Value	Equation (7)
$f_{4}$	Ch	Chroma	Equation (8)
$f_{5}$	$\sigma_{Ch}^{2}$	Variance of chroma	Equation (9)
$f_{6}$	CF	Colorfulness	Equation (11)
$f_{7}$	$\sigma_{I}^{2}$	Sharpness	Equation (14)
$f_{8}$	HD	Hue disparity	Equation (17)
$f_{9}$	IE	Image entropy	Equation (16)

**Table 3:** Summary of terminologies and derivations for evaluating the hazy/haze-free image classification task.

True positive TP	False positive FP
Given: hazy images	Given: haze-free images
Predicted: hazy	Predicted: hazy
**False negative FN**	**True negative TN**
Given: hazy images	Given: haze-free images
Predicted: haze-free	Predicted: haze-free

**Table 4:** The accuracy report for the hazy/haze-free image classification task using haze density evaluators.

Class	FADE	DF	${\mathrm{HDE}}_{\beta }$
DV	0.9866	0.2968	0.4725
*P*	1921		
TP	1785	1672	1750
TPR	92.9%	87.0%	91.1%
FN	136	249	171
FNR	7.1%	13.0%	8.9%
*N*	2168		
TN	2005	2038	2084
TNR	92.5%	94.0%	96.1%
FP	163	130	84
FPR	7.5%	6.0%	3.9%
$ACC_{\Phi }$	92.7%	90.7%	93.8%

**Table 5:** The updated accuracy report for hazy/haze-free image classification task using haze density evaluators.

Class	FADE	DF	${\mathrm{HDE}}_{\beta }$	HDE
DV	0.9866	0.2968	0.4725	0.8811
*P*	1921			
TP	1785	1672	1750	1843
TPR	92.9%	87.0%	91.1%	95.9%
FN	136	249	171	78
FNR	7.1%	13.0%	8.9%	4.1%
*N*	2168			
TN	2005	2038	2084	2081
TNR	92.5%	94.0%	96.1%	96.0%
FP	163	130	84	87
FPR	7.5%	6.0%	3.9%	4.0%
$ACC_{\Phi }$	92.7%	90.7%	93.8%	96.0%

**Table 6:** Quantitative evaluation results of dehazing algorithms on LIVE and D-HAZY datasets. NA stands for not available.

	Algorithm	He et al. [6]	Tarel and Hautiere [8]	Zhu et al. [49]
Dataset				
LIVE	assessed by	0.1882	0.2172	0.2605
D-HAZY	HDE	0.2925	0.3739	0.3674
LIVE	assessed by	0.8700	0.7480	1.0480
	FADE [50]			
D-HAZY	assessed by	0.8110	0.7190	NA
	SSIM [42]			

**Table 7:** The quantitative evaluation results of different dehazing methods on the IVC, D-HAZY, O-HAZE, and I-HAZE datasets.

Dataset		IVC		D-HAZY		O-HAZE		I-HAZE	
	**Metric**	***e***	***r***	**TMQI**	**FSIMc**	**TMQI**	**FSIMc**	**TMQI**	**FSIMc**
**Method**									
Tarel and Hautiere [8]		1.30	2.15	0.8000	0.8703	0.8416	0.7733	0.7740	0.8055
He et al. [6]		0.39	1.57	0.8631	0.9002	0.8403	0.8423	0.7319	0.8208
Zhu et al. [49]		0.78	1.17	0.8206	0.8880	0.8118	0.7738	0.7512	0.8252
HDE-based algorithm		1.04	1.57	0.8564	0.8621	0.8340	0.8218	0.7677	0.8517

**Table 8:** Run-time in seconds of different haze density evaluators for various image sizes.

Evaluator	Image Size				
	640 × 480	800 × 600	1024 × 768	1920 × 1080	4096 × 2160
FADE	0.45	0.73	1.11	2.84	12.36
DF	0.10	0.18	0.27	0.70	3.12
HDE	0.07	0.12	0.18	0.41	2.06

## Data Availability

Data available in a publicly accessible repository. The data presented in this study are openly available in [36,37,38,40,41,42,58] and FigShare at [10.6084/m9.figshare.14729001.v1] and [10.6084/m9.figshare.14729052.v1].

## Appendix A

This appendix provides details about the derivation of the scene radiance’s features in Equations (21)–(23). Firstly, the scene radiance’s dark channel is derived while using Equation (3), as follows:

(A1) $$\begin{array}{ccc} J^{dark}\left(x\right) & = & \min_{y\in \Omega (x)}\left[\min_{c\in \{R,G,B\}}J^{c}\left(y\right)\right], \end{array}$$

(A2) $$\begin{array}{ccc} & = & \min_{y\in \Omega (x)}\left\{\min_{c\in \{R,G,B\}}\left[\frac{I^{c}\left(y\right)-A^{c}}{t(y)}+A^{c}\right]\right\}, \end{array}$$

(A3) $$\begin{array}{ccc} & = & \min_{y\in \Omega (x)}\left\{\frac{\min_{c\in \{R,G,B\}}I^{c}\left(y\right)-A}{t(y)}+A\right\}, \end{array}$$

(A4) $$\begin{array}{ccc} & = & \frac{\min_{y\in \Omega (x)}\left[\min_{c\in \{R,G,B\}}I^{c}\left(y\right)\right]-A}{t(x)}+A, \end{array}$$

(A5) $$\begin{array}{ccc} & = & A-\frac{A-I_{m\Omega }\left(x\right)}{t(x)}, \end{array}$$

where $I_{m\Omega }\left(x\right)=\min_{y\in \Omega (x)}\left[\min_{c\in \{R,G,B\}}I^{c}\left(y\right)\right]$. In the above equations, two assumptions regarding the transmission map and atmospheric light in Section 3.4 have been exploited to simplify the derivation of $J^{dark}$. Regarding the transmission map, since it is a single channel variable, there is no superscript *c* associated with *t*, leading to $\min_{c\in \{R,G,B\}}t\left(y\right)=t\left(y\right)$, as in the transformation from Equation (A2) to Equation (A4). The assumption regarding its homogeneity in a local patch then results in $\min_{y\in \Omega (x)}t\left(y\right)=t\left(x\right)$, which explains the transformation from Equation (A4) to Equation (A5). Simply put, the minimum value of the transmission map in a local patch Ω(x) centered at *x* is t(x) itself because it remains unchanged within Ω(x). Regarding the atmospheric light, since it is assumed that $A^{R}=A^{G}=A^{B}=A$, the transformation from Equation (A3) to Equation (A4) is explicable. Additionally, because the atmospheric light is constant over the entire image, no spatial coordinates are attached to it. This image-wise fixity also leads to $\min_{y\in \Omega (x)}A=A$, as in the transformation from Equation (A4) to Equation (A5).

Using Equation (7), the product of saturation and value of the scene radiance is given, as follows:

(A6) $$\begin{array}{ccc} SV[J(x)] & = & \max_{c\in \{R,G,B\}}J^{c}\left(x\right)-\min_{c\in \{R,G,B\}}J^{c}\left(x\right), \end{array}$$

(A7) $$\begin{array}{ccc} & = & \max_{c\in \{R,G,B\}}\left[\frac{I^{c}\left(x\right)-A^{c}}{t(x)}+A^{c}\right]-\min_{c\in \{R,G,B\}}\left[\frac{I^{c}\left(x\right)-A^{c}}{t(x)}+A^{c}\right], \end{array}$$

(A8) $$\begin{array}{ccc} & = & \left[\frac{\max_{c\in \{R,G,B\}}I^{c}\left(x\right)}{t(x)}-\frac{A}{t(x)}+A\right]-\left[\frac{\min_{c\in \{R,G,B\}}I^{c}\left(x\right)}{t(x)}-\frac{A}{t(x)}+A\right], \end{array}$$

(A9) $$\begin{array}{ccc} & = & \frac{1}{t(x)}\left[\max_{c\in \{R,G,B\}}I^{c}\left(x\right)-\min_{c\in \{R,G,B\}}I^{c}\left(x\right)\right], \end{array}$$

(A10) $$\begin{array}{ccc} & = & \frac{I_{mc}\left(x\right)}{t(x)}, \end{array}$$

where $I_{mc}\left(x\right)=\max_{c\in \{R,G,B\}}I^{c}\left(x\right)-\min_{c\in \{R,G,B\}}I^{c}\left(x\right)$.

Finally, the derivation of the scene radiance’s sharpness is self-explicable when considering it from a statistical perspective. Because the image sharpness is defined using the measure of variability, as in Equations (14) and (15), the patch-wise fixity of the transmission map, the image-wise fixity of the atmospheric light, and the linearity of Equation (20) altogether explain the derivation of the scene radiance’s sharpness.

## Appendix B

This appendix provides the experimental results of using the decision value that was determined in this study to classify new hazy/haze-free images. In this context, these images are not in the datasets summarized in Table 1. Instead, they come from two testing sets of the RESIDE dataset [58] and they consist of 1020 hazy images and 552 haze-free images. The results that are tabulated in Table A1 demonstrate that the proposed HDE is still the best performing method, followed by the FADE and DF in descending order. Furthermore, the source code and relevant data are publicly available at https://github.com/v1t0ry/Haziness-degree-evaluator (accessed on May 8th, 2021) for reproducing the reported results.

**Table A1:** Accuracy report for hazy/haze-free image classification task using haze density evaluators on new data.

Class	FADE	DF	HDE
DV	0.9866	0.2968	0.8811
*P*	1020		
TP	863	242	929
TPR	84.6%	23.7%	91.1%
FN	157	778	91
FNR	15.4%	76.3%	8.9%
*N*	552		
TN	446	508	507
TNR	80.8%	92.0%	91.9%
FP	106	44	45
FPR	19.2%	8.0%	8.1%
$ACC_{\Phi }$	83.3%	47.7%	91.4%
